# Structure of HLA-A*0301 in complex with a peptide of proteolipid protein: insights into the role of HLA-­A alleles in susceptibility to multiple sclerosis

**DOI:** 10.1107/S0907444911007888

**Published:** 2011-04-13

**Authors:** Róisín M. McMahon, Lone Friis, Christian Siebold, Manuel A. Friese, Lars Fugger, E. Yvonne Jones

**Affiliations:** a Medical Research Council Human Immunology Unit, Weatherall Institute of Molecular Medicine, John Radcliffe Hospital, University of Oxford, Oxford OX3 9DS, England; b Division of Structural Biology, The Henry Wellcome Building for Genomic Medicine, University of Oxford, Roosevelt Drive, Oxford OX3 7BN, England; c Department of Clinical Neurology, Level 6, West Wing, John Radcliffe Hospital, University of Oxford, Oxford OX3 9DU, England; d Institut für Neuroimmunologie und Klinische MS-Forschung, Zentrum für Molekulare Neurobiologie, Universitätsklinikum Hamburg-Eppendorf, Falkenried 94, 20251 Hamburg, Germany; e Clinical Institute, Aarhus University Hospital, Skejby Sygehus, Brendstrupgaardsvej 100, 8200 N Aarhus, Denmark

**Keywords:** HLA-A*0301, MHC, multiple sclerosis, autoimmune disease

## Abstract

The structure of the human major histocompatability (MHC) class I molecule HLA-A*0301 (HLA-A3) in complex with a nonameric peptide (KLIETYFSK) has been determined by X-­ray crystallography to 2.7 Å resolution. HLA-A3 is a predisposing allele for multiple sclerosis (MS), an auto­immune disease of the central nervous system. The KLIETYFSK peptide is a naturally processed epitope of proteolipid protein, a myelin protein and candidate target for immune-mediated myelin destruction in MS. Comparison of the structure of HLA-A3 with that of HLA-A2, an MHC class I molecule which is protective against MS, indicates that both MHC class I molecules present very similar faces for T-cell receptor recognition whilst differing in the specificity of their peptide-binding grooves. These characteristics may underlie the opposing (predisposing *versus* protective) associations that they exhibit both in humans and in mouse models of MS-­like disease. Furthermore, subtle alterations within the peptide-binding groove of HLA-A3 and other A3-like MHC class I molecules, members of the so-called A3 superfamily, may be sufficient to alter their presentation of autoantigen peptides such as KLIETYFSK. This in turn may modulate their contribution to the associated risk of autoimmune disease.

## Abstract 2

The structure of the human major histocompatability (MHC) class I molecule HLA-A*0301 (HLA-A3) in complex with a nonameric peptide (KLIETYFSK) has been determined by X-­ray crystallography to 2.7 Å resolution.

## 1. Introduction

Major histocompatability (MHC) class I molecules are heterodimeric glycoproteins that are present on the surface of almost all nucleated cells. Their primary role is to present peptides derived from endogenous cellular proteins for recognition by T-cell receptors (TCRs) on the surface of CD8^+^ cytotoxic T-lymphocytes (CTLs). In this manner, MHC class I molecules contribute to an immune surveillance system in which TCRs monitor the population of presented peptides for indications of cellular infection or transformation. TCR recognition of an MHC class I molecule-presented nonself-peptide or altered self-peptide induces CD8^+^ CTL-mediated destruction of the target cell and as such MHC class I molecules are at the forefront of adaptive immune defences (Guermonprez *et al.*, 2002 ▶).

The first atomic level structure of an MHC class I molecule was reported for HLA-A2 (Bjorkman *et al.*, 1987 ▶; Saper *et al.*, 1991 ▶). Subsequently, structures of various MHC class I allelic products in complex with various specific peptides have been reported. Each MHC class I structure has revealed an architecture fundamentally similar to that of HLA-A2. Briefly, the α chain forms two membrane-distal domains (α1 and α2) which together generate a solvent-exposed groove composed of an eight-antiparallel-β-strand platform bordered on either long edge by an α-helix. The membrane-proximal immuno­globulin-like α3 domain of the α chain associates non­covalently with the invariant immunoglobulin-like β_2_-microglobulin (β_2_m). Peptides (typically 8–10 amino acids in length) bind within the groove, stabilized by a network of conserved hydrogen-bond interactions and allele-specific contacts. Polymorphisms in the residues lining the groove influence the preference that each MHC groove has for particular peptide motifs, defined as preferences for particular anchor residues whose side chains bind in specific pockets within the groove. Individual HLA alleles have been grouped into HLA superfamilies on the basis of commonalities in these peptide-motif preferences (Sidney *et al.*, 2008 ▶; Sette & Sidney, 1999 ▶).

Multiple sclerosis (MS) is an autoimmune disease of the central nervous system that is characterized by demyelination and neuronal and axonal degeneration. The disease arises from an autoreactive T-cell mediated immune response directed against healthy myelin, the lipid-protein sheath which encases neuronal axons (reviewed by Sospedra & Martin, 2005 ▶). MS is genetically associated with the HLA region on chromosome 6, which is consistent with a role for MHC molecules in presentation of myelin-associated peptides to potentially autoreactive T cells. Whilst MHC class II alleles (in particular those of the DR2 haplotype: HLA-DRB5*0101, HLA-DRB1*0501 and HLA-DQB1*0601) remain the strong­est identified associated genetic risk factor for MS, there is evidence for an independent association with the MHC class I molecule-encoding HLA-A region. More specifically, HLA-A3 approximately doubles disease risk, whilst HLA-A2 exerts a dominant protective effect, approximately halving the relative risk conferred by either HLA-A3 or the DR2 haplotype (Harbo *et al.*, 2004 ▶; Fogdell-Hahn *et al.*, 2000 ▶).

Animal models of MS-like disease have helped to uncover complexities in the opposing actions of HLA-A3 and HLA-A2 in MS. Transgenic mice expressing either or both of these MHC class I alleles were crossed with a mouse transgenic for a MS patient-derived T-cell receptor that recognizes a peptide from proteolipid protein (PLP residues 45–53; KLIETYFSK) presented by HLA-A3 molecules (Honma *et al.*, 1997 ▶); proteolipid protein is a major component of myelin and a candidate target for immune-mediated myelin destruction (Sospedra & Martin, 2005 ▶). These studies showed that the transgenic CTLs recognizing HLA-A3/PLP45-53 are sufficient to initiate an early mild form of MS-like disease, whilst co-expression of HLA-A2 as an additional transgene significantly moderated disease in the same mice. This protection resulted mainly from the deletion of most of the autoreactive CTLs in the thymus and partly from down-regulation of surface autoreactive TCR expression (Friese *et al.*, 2008 ▶).

Notably, in contrast to HLA-A3, other members of the A3 superfamily (HLA-A11, HLA-A3301, HLA-A3101 and HLA-Aw*6801), which by definition share peptide-binding properties with HLA-A3, do not appear to be associated with MS (Harbo *et al.*, 2004 ▶; Kheradvar *et al.*, 2004 ▶). A report of a modest predisposing effect of HLA-A11 in the Indian population was tempered by the particular small sample group studied (Kankonkar *et al.*, 2003 ▶). In order to address the possible molecular determinants of the opposing disease associations of HLA-A3 and HLA-A2 and the neutrality of other A3-superfamily members, we solved the crystal structure of HLA-A3 in complex with the autoantigen peptide PLP45-53 (KLIETYFSK). This structure provides the first experimentally derived crystallographic view of the HLA-A3 peptide-binding groove, allowing detailed comparison with previously reported structures of other members of the A3 superfamily. The HLA-A3/PLP45-53 structure also allows us to assess the extent of structural equivalence between the surface exposed for TCR recognition in HLA-A2 and in HLA-A3; a potentially important contributing factor to the frequency of TCR cross-reactivity to HLA-A2 and HLA-A3 peptide complexes.

## 2. Materials and methods

### 2.1. Cloning

cDNA of HLA-A*0301 α chain was derived by reverse transcription of mRNA isolated from an HLA-A*0301 positive donor and DNA encoding residues 1–274 was amplified by polymerase chain reaction. The final HLA-A*0301 sequence was inserted into the pET22b+ vector (Novagen) and verified by sequencing.

### 2.2. Protein production and purification

Recombinant β_2_m and HLA*A301 α chain were produced as described previously (Friese *et al.*, 2008 ▶). Briefly, β_2_m and HLA*A301 α chain were expressed in *Escherichia coli* BLR (DE3) cells (Novagen) as inclusion bodies, which were subsequently isolated, washed in Triton X-­100 and resolubilized in 8 *M* urea, 10 m*M* NaH_2_PO_4_, 10 m*M* Tris pH 8.0, 0.1 m*M* EDTA, 0.1 m*M* DTT. Equal amounts of β_2_m and HLA*A301 α chain were then refolded together with PLP45-53 peptide (ratio 3:3:1) by rapid dilution in 100 m*M* Tris pH 8.0, 2 m*M* EDTA, 400 m*M* l-arginine–HCl, 5 m*M* reduced glutathione, 0.5 m*M* oxidized glutathione for 72 h at 277 K. Refolded MHC molecules were purified by size-exclusion chromatography on a Superdex 75pg HiLoad 26/60 column (GE Healthcare) in 20 m*M* Tris pH 8.0, 0.1 *M* NaCl, concentrated and then frozen at 193 K in aliquots. Prior to crystallization, the A3–PLP45-53 complexes were further purified by anion-exchange chromatography on a 5 ml HiTrap QFF column (GE Healthcare) eluting with a salt gradient to 1 *M* NaCl in 20 m*M* Tris pH 8.0. Purified protein was exchanged into 10 m*M* NaCl, 10 m*M* HEPES pH 7.0 and concentrated to 6.9 mg ml^−1^.

### 2.3. Peptide production and purification

The PLP45-53 (KLIETYFSK) peptide used to generate HLA-A3 complexes was purchased from Schafner-N (Copenhagen) as a >99% HPLC-purified preparation synthesized using FMOC chemistry.

### 2.4. Crystallization and data collection

Crystallization experiments were set up at 293 K using a Cartesian Technologies Microsys Mic4000 (Walter *et al.*, 2005 ▶) with 100 nl protein solution (6.9 mg ml^−1^ in 10 m*M* NaCl, 10 m*M* HEPES pH 7.0) and 100 nl reservoir solution. Needle-like crystals [average dimensions of 20–30 µm (width) × 230–270 µm (length) × 10–15 µm (depth)] were grown by the sitting-drop vapour-diffusion method against a reservoir consisting of 20%(*w*/*v*) polyethylene glycol 3350, 0.1 *M* Bis-Tris propane pH 8.5 and 0.2 *M* sodium/potassium phosphate (Molecular Dimensions PACT Premier condition No. 94) and used directly for data collection. A single-crystal was cryoprotected in 25% ethylene glycol and vitrified at 100 K. A complete data set to 2.7 Å was collected at the Diamond Light Source, Oxfordshire, England on beamline I04 (beam diameter of 80–100 µm). The data were processed and scaled with the *HKL*-2000 suite (Otwinowski & Minor, 1997 ▶). The crystal belonged to space group *P*2_1_2_1_2_1_, with unit-cell parameters *a* = 62.9, *b* = 65.5, *c* = 107.3 Å, α = β = γ = 90°. There is one molecule in the asymmetric unit.

### 2.5. Structure determination and refinement

The structure of A3–PLP45-53 was solved by molecular replacement with *MOLREP* (Vagin & Teplyakov, 2010 ▶) using the structure of HLA-A2 with the peptide omitted (PDB entry 1duz; Khan *et al.*, 2000 ▶) as a search model. Validating the molecular-replacement solution, the initial *F*_o_ − *F*_c_ difference map contoured at 3σ showed clear electron density along the peptide-binding groove corresponding to the PLP45-53 peptide. Several rounds of manual inspection and rebuilding using *Coot* (Emsley & Cowtan, 2004 ▶) were performed interspersed by restrained refinement in *REFMAC*5 (Murshudov *et al.*, 2011 ▶). This included manual building of the PLP45-53 (KLIETYFSK) peptide. The *PHENIX* software suite (Adams *et al.*, 2002 ▶) was then employed for final rounds of individual ADP and TLS refinement. Groups for TLS were defined using the *TLSMD* server (Painter & Merritt, 2006 ▶). The model was refined to con­vergence against 95% of the data to a final *R* factor of 19% and *R*_free_ of 25%. Full details of crystallization, data collection, processing and refinement can be found in Table 1 ▶. The final structure contains the HLA-A*0301 α chain residues 2–274, the β_2_m residues 1–98, KLIETYFSK peptide and 34 water molecules.

### 2.6. Structure superposition and graphical representations

All superpositions of atomic structures were performed using *SHP* (Stuart *et al.*, 1979 ▶). All graphical representations of atomic models were generated with *PyMOL* (http://www.pymol.org).

### 2.7. Model quality and analysis

The final model was validated using *MolProbity* (Chen *et al.*, 2010 ▶) and interactions between the peptide and binding groove were analysed using *LIGPLOT* (Wallace *et al.*, 1995 ▶).

## 3. Results and discussion

**Figure 1:**
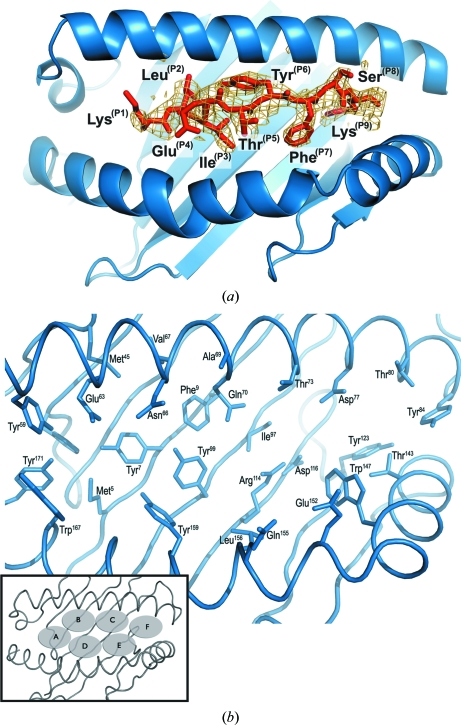
The crystal structure of A3–PLP45-53. (*a*) The view is from above into the binding groove of A3–PLP45-53. The MHC heavy chain is shown in blue and the peptide PLP45-53 in red. σ_A_-weighted *F*_o_ − *F*_c_ electron density for the peptide PLP45-53 (generated in *PHENIX* after deletion of the peptide followed by simulated annealing to minimize model bias) is also shown contoured at 2.7σ. (*b*) Characteristics of the HLA-A3 peptide-binding groove. View from above into the binding groove of HLA-A3. The side chains of individual MHC heavy-chain residues involved in peptide binding are shown as sticks and labelled. The schematic inset provides a key to the locations of the canonical pockets A–F.

The structure of PLP45-53 in complex with HLA-A3 (A3–PLP45-53) was determined at 2.7 Å resolution to an *R* factor of 19.3% (*R*_free_ = 25%). Details of data collection and additional indicators of the quality of the model are given in Table 1 ▶. The architecture of A3–PLP45-53 is typical of all previous MHC class I structures (Fig. 1 ▶ *a*) and the peptide-binding groove can be described in terms of the six classic MHC class I molecule pockets A–F (nomenclature as defined in Saper *et al.*, 1991 ▶; see inset in Fig. 1 ▶ *b*). PLP45-53, a nonameric peptide, adopts a typical arched conformation within this groove with its N- and C-terminal residues bound in the A and F pockets, respectively (Fig. 1 ▶). MHC residues implicated in binding PLP45-53 are illustrated in Fig. 1 ▶(*b*) and their interactions are listed in Table 2 ▶.

### 3.1. Characteristics of the HLA-A3 peptide-binding groove

The canonical hydrogen-bond network between nonpolymorphic A-pocket residues and the main-chain atoms of the peptide N-terminus is conserved in A3–PLP45-53 (Guo *et al.*, 1992 ▶; Silver *et al.*, 1992 ▶), Additional hydrophobic interactions between the aliphatic portion of the Lys side chain and the ring of Trp167 further stabilize the peptide position 1 (P1) Lys within the pocket (Table 2 ▶).

The B pocket is a primary peptide anchor-binding pocket in HLA-A3 (Maier *et al.*, 1994 ▶; Falk *et al.*, 1991 ▶) alleles. The most significant residues influencing the character and thus peptide side-chain binding specificity of the B pocket of HLA-A3 are Tyr7, Phe9, Met45, Glu63, Asn66, Val67, Gln70 and Tyr99. The pocket has a predominantly hydrophobic character consistent with the reported preference for Val, Leu and Met at position 2 of the peptides which bind to HLA-A3 (Maier *et al.*, 1994 ▶). PLP P2 Leu tucks under Asn66 with its side chain directed towards the α1 helix and its main chain involved in a hydrogen bond to Glu63 (Table 2 ▶). However, it does not occupy the entire volume available, which is consistent with the ability of this pocket to also accommodate a larger Met side chain.

P3 Ile sits snugly within the D pocket with its side chain orientated parallel to the α2 helix and its main chain involved in a hydrogen bond to Tyr99. Hydrophobic interactions with Tyr159 and Tyr99 further stabilize binding. There is a very pronounced arch in the peptide backbone from P3 to P4 that is typical of nonameric peptides bound to MHC class I molecules (Madden *et al.*, 1991 ▶). As a result, both P4 Glu and P5 Thr are solvent-exposed and extend above the binding groove, positioning them as potential TCR contact residues. Neither of these peptide residues makes any contacts to MHC heavy-chain residues and there is insufficient density to unambiguously model the position of the P4 Glu side chain, which is consistent with some flexibility (Fig. 1 ▶ *a*). The groove opens beneath the main-chain atoms of P6 Tyr to form the C pocket. Rather than dock into this available volume, the aromatic ring of P6 Tyr is positioned approximately parallel to the α1 helix and directed towards the N-terminus of the peptide, where it is stabilized by a hydrogen bond to Asn66 and hydrophobic interactions with residues Ala69, Thr73 and Gln70. A water molecule occupies the remaining volume of pocket C co­ordinated by Tyr99, Glu70 and the hydroxyl group of P6 Tyr. In contrast to residues P4–P6, the P7 Phe side chain is deeply buried within the groove inserting into the E pocket. The character of this pocket is predominantly hydrophobic, with Leu156 at its base and Trp147 contributing to its border. P7 Phe is stabilized within this pocket *via* hydrophobic stacking of its aromatic ring against the alkyl stalk of the Gln152 side chain and by additional hydrophobic interactions with Tyr147. The hydroxyl group of P8 Ser is entirely solvent-exposed. A main-chain hydrogen bond to Trp147 and an additional side-chain hydrogen bond to Lys146 help to stabilize this residue within the groove.

The F pocket of HLA-A3 is identical to that of HLA-A11, which shares all of the same polymorphic residues at the C-­terminal binding site (Li & Bouvier, 2004 ▶). The canonical hydrogen-bond network between nonpolymorphic F-pocket residues and main-chain atoms of the peptide C-terminus is conserved in A3–PLP45-53 (Guo *et al.*, 1992 ▶; Silver *et al.*, 1992 ▶). P9 Lys is further stabilized by a triad of aspartic acid residues (residues 74, 77 and 116) which serve to anchor the positively charged peptide side chain within the pocket.

### 3.2. Comparison of A3–PLP45-53 with other members of the A3 superfamily

Across the A3-superfamily the architecture of the B pocket is similar, reflecting their overlapping peptide-binding repertoires (Sidney *et al.*, 1996 ▶). Residues 7, 45, 66, 67 and 99 are conserved family wide. Residues 63 and 70 are polymorphic, but Asn (HLA-A33 and HLA-Aw*68) to Glu (HLA-A31, HLA-A3 and HLA-A11) and Gln (HLA-A3, HLA-A11 and HLA-Aw*68) to His (HLA-A31 and HLA-A33) substitutions retain potential hydrogen-bonding forming residues at these positions. Residue 9, which is known to critically influence side-chain preference in the B pocket, is a Phe in HLA-A3. In the other A3-superfamily members residue 9 is a Tyr and whilst maintaining the same constraint on the volume of the pocket, the extra hydrogen-bonding potential may contribute to the ability of HLA-A11, HLA-A31, HLA-A33 and HLA-Aw*68 to accommodate a polar Thr side chain as well as more hydrophobic anchors (Guo *et al.*, 1992 ▶; Falk *et al.*, 1994 ▶; Kubo *et al.*, 1994 ▶).

Across the A3 superfamily, F-pocket residues 74, 77 and 116 are strictly conserved aspartic acid residues. Consequently, this negatively charged pocket has a preference for positively charged Arg and Lys side chains. Notably, however, individual A3-family submembers exhibit distinct preferences for either an Arg or a Lys at position 9 (Sidney *et al.*, 1996 ▶). Residue 97 is known to be critical in determining the depth of the F pocket in HLA-A3-like molecules (Li & Bouvier, 2005 ▶) and to influence the choice of Arg or Lys at this position. In HLA-A3, an Ile at position 97 is consistent with the preferred binding of Lys in the F pocket (Kubo *et al.*, 1994 ▶). The branched side chain of Ile97 narrows the available space in this part of the groove, favouring accommodation of the Lys side chain rather than the longer alkyl chain of Arg. Greater space is provided for the latter to bind by a Met at position 97 in HLA-Aw*68, HLA-A31 and HLA-A33.

**Figure 2:**
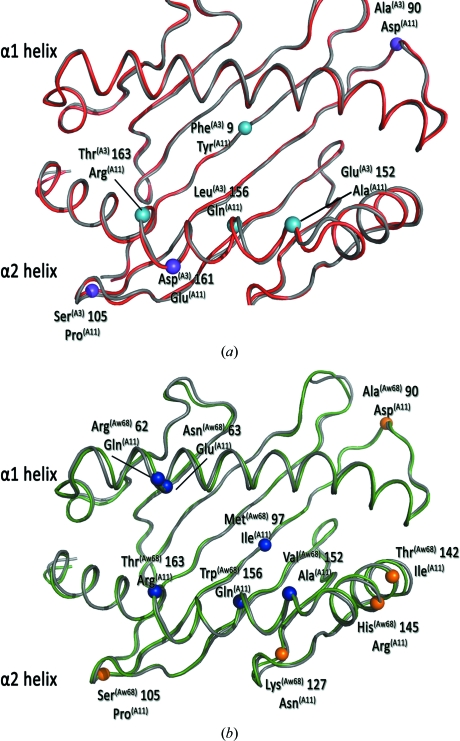
Structural comparison of HLA-A11 with HLA-A3 and HLA-Aw*68. (*a*) The C^α^ atoms of residues that differ between HLA-A11 (grey) and HLA-A3 (red) are represented as small spheres, labelled and coloured cyan if the side chains point into the binding groove and purple if they do not. The α1 and α2 helices are as indicated. (*b*) The C^α^ atoms of residues that differ between HLA-A11 (grey) and HLA-Aw*68 (green) are represented as small spheres, labelled and coloured blue if the side chains point into the binding groove in HLA-A3 and orange if they do not. The α1 and α2 helices are as indicated.

Whilst HLA-A3 is known to confer an enhanced relative risk of MS, a sister allele of the A3 superfamily, HLA-A11 (A11), has no notable association with MS risk. This difference in associated risk is striking as HLA-A3 and HLA-A11 differ by only seven residues in the extracellular region of their heavy chain, of which only four residues (9, 152, 156 and 163) influence the peptide-binding groove (Fig. 2 ▶ *a*).

Detailed comparison of the structure of A3–PLP45-53 with that of HLA-A11 presenting two nonamer peptides from HIV reverse transcriptase (RT313-321) and SARS nucleocapsid protein (SNP362–370) [PDB entries 1q94 (Li & Bouvier, 2004 ▶) and 1x7q (Blicher *et al.*, 2005 ▶), respectively] indicates that one polymorphic residue, 152 (Fig. 2 ▶ *a*), is sufficient to alter peptide binding between these two MHC class I molecules. Glu152 (A3) and Ala152 (A11) make differing contacts to the respective P7 residue which correlates with the absence (A3/PLP45-53) and presence (A11/RT313-321 and A11/SNP362-370) of a secondary anchor respectively at the adjacent P6 residue, influencing how deeply each peptide binds within the groove.

As with HLA-A11, HLA-Aw*68, another sister allele of the A3 superfamily, has no known association with MS. HLA-Aw*68 differs from HLA-A3 at 16 residues in its heavy chain and thus at the level of its amino-acid sequence is less similar to HLA-A3 than HLA-A11. Of the six polymorphic residues between HLA-A11 and HLA-Aw*68 (Fig. 2 ▶ *b*) with the potential to alter peptide binding (62, 63, 97, 152, 156 and 163), the most significant alterations between the two alleles are again in the central section of the binding groove. In HLA-Aw*68, bulkier residues at 97, 152 and 156 (Met, Val and Trp) make the groove shallower and narrower in the vicinity of the C and E pockets relative to HLA-A11, where these residues are Ile, Ala and Glu, respectively. Consequently, HLA-Aw*68 and HLA-A11 may be predicted to bind the same peptides because of the shared primary anchor preferences, but can be expected to present them in differing manners. Supporting this, the nonamer peptides RT313-321 and Nef73-82 both bind with high affinity to HLA-A11 (and HLA-A3) but bind considerably more weakly to HLA-Aw*68. Whilst members of the A3 superfamily can have similar peptide-binding repertoires, polymorphic differences can modify the nature and stability of the MHC–peptide inter­action (Li & Bouvier, 2005 ▶), which in turn has implications for TCR inter­actions. HLA-B2705 and HLA-B2709 (Hülsmeyer *et al.*, 2002 ▶), which are disease-associated and non-associated for spondyloarthropathies, respectively, have previously illustrated the power of a single amino-acid alteration to modulate disease association of alleles presenting a common peptide. In the case of the HLA-A3 superfamily, subtle alterations among the binding grooves of HLA-A3, HLA-A11 and HLA-Aw*68 could potentially have significant implications for peptide presentation of autoantigen peptides such as PLP45-53 and hence their associated disease risk.

### 3.3. HLA-A3 and HLA-A2 differ in the architecture of their binding grooves but present the same HLA surface for TCR recognition

**Figure 3:**
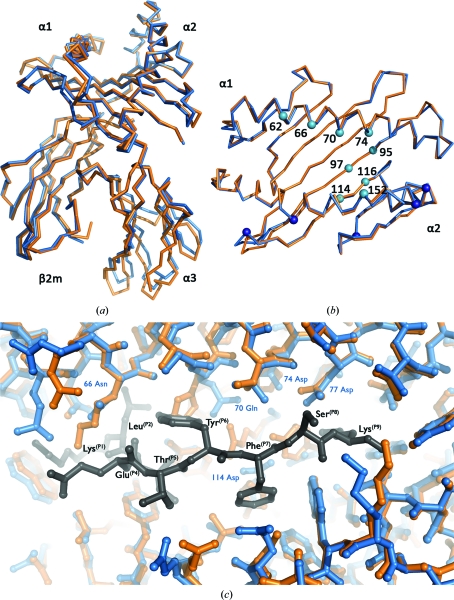
Structural comparison of HLA-A3 and HLA-A2. (*a*) Superposition of A3–PLP45-53 (blue) and HLA-A2 (orange). HLA-A3 and HLA-A2 show a high degree of equivalence in their α1, α2 and β2m domains. The α3 domains of HLA-A3 and HLA-A2 are also very structurally similar, but the relative position of this domain is subject to a rigid-body shift between molecules. (*b*) The C^α^ atoms of residues that differ between HLA-A3 and HLA-A2 are represented as small spheres and coloured cyan if the side chains point into the binding groove in HLA-A3 and dark blue if they do not. The α1 and α2 helices are labelled. (*c*) Viewed from above the binding groove, PLP45-53 (grey) is shown bound to HLA-A3 (blue) and superposed upon the structure of HLA-A2 (orange). The groove is orientated as in Fig. 3 ▶(*b*). Polymorphic HLA-A3 residues that differ from HLA-A2 and that contribute to peptide binding are labelled.

Superposition of the structure of HLA-A3–PLP45-53 with the previously reported crystal structure of HLA-A2 (PDB entry 1duz) indicates that the overall architecture of the HLA-A3 peptide-binding groove is essentially identical to that of HLA-A2 (0.5 Å r.m.s.d. for superposition of the 179 equivalent pairs of C^α^ atoms of the α1 and α2 domains). The position of the α3 domain relative to the binding groove and β2m is subject to a small rigid-body shift between HLA-A3 and HLA-A2, but the individual α3 domains are essentially identical (0.36 Å r.m.s.d. for superposition of 96 equivalent pairs of C^α^ atoms; Fig. 3 ▶ *a*). This is consistent with previous comparisons of the isolated MHC class I molecules in which the α3 domain was observed to vary in its position relative to the rest of the molecule by up to 3.5° (Madden, 1995 ▶).

The heavy chains of HLA-A3 and HLA-A2 differ by only 19 amino acids (93% shared sequence identity). These polymorphic residues map primarily to the binding groove, where the specific nature and orientation of the side chain confers a distinct character on the respective grooves (Table 3 ▶, Figs. 3 ▶ *b* and 3 ▶ *c*).

HLA-A3 and HLA-A2 exhibit overlapping preferences for P2 anchors in the B pocket (Val, Leu and Met in HLA-A3; Leu and Met in HLA-A2; Kubo *et al.*, 1994 ▶; Falk *et al.*, 1991 ▶; Table 3 ▶). Of the residues contributing to the B pocket, only position 66 differs between HLA-A3 and HLA-A2 (Asn and Lys, respectively). This substitution does not affect HLA-A3 binding of P2 Leu, which is positioned under Asn66 pointing towards the α1 helix (Fig. 3 ▶ *c*), nor does it alter the character of the pocket, which remains predominantly hydrophobic.

The majority of structural differences between the binding grooves of HLA-A3 and HLA-A2 are within the mid-section of the groove, arising from polymorphisms at positions 70, 97, 114 and 116 (Figs. 3 ▶ *b* and 3 ▶ *c*). Ile97 (Arg in HLA-A2) generates a more open C-pocket in HLA-A3 relative to HLA-A2, whilst Gln70 (His in HLA-A2) leaves space to accommodate the P6 Tyr side chain, which is directed diagonally towards the α1 helix in A3–PLP45-53. Substitution of Tyr (the residue in HLA-A2) by Asp at position 116 in HLA-A3 opens the base of the F pocket, providing a sufficient volume to accommodate P9 Lys which would be sterically hindered in HLA-A2. In addition, the F pocket of HLA-A2 is much more hydrophobic than HLA-A3, reflecting its preference for Val at P9 (Table 3 ▶).

Whilst their respective grooves vary markedly, there are notably no residue differences between HLA-A3 and HLA-A2 which are directly exposed at the TCR recognition surface on the α1 and α2 helices (Fig. 3 ▶ *b*) Therefore, HLA-A3 and HLA-A2 present structurally identical surfaces for TCR engagement. Conversely, the differing internal architecture of the binding grooves is consistent with HLA-A3 and HLA-A2 presenting a divergent pool of peptides. However, the exposed features of these peptides could in some cases be similar (as observed for some peptide–MHC class II structures; Lang *et al.*, 2002 ▶). Thus, the similarity in surface contribution for TCR recognition by HLA-A3 and HLA-A2 suggests the possibility of structural mimicry occurring between HLA-A3 peptide and HLA-A2 peptide complexes, providing the potential to promote TCR cross-reactivity and to modify thymic selection.

Among HLA class I alleles, HLA-A2 is the commonest worldwide and its high frequency in most populations suggests that it is a particularly important player in protective T-cell mediated immunity. This could reflect either the ability to present (i) a single dominant epitope from a ubiquitous pathogen, (ii) some peptide sequence(s) widely conserved among microbial species or (iii) some special versatility in its peptide-presenting abilities, possibly because of its somewhat indiscriminate preferences for hydrophobic anchor residues. Indeed, it presents epitopes from such globally important pathogens as influenza, Epstein–Barr and hepatitis viruses as well as malaria (Browning & Krausa, 1996 ▶). Possibly, it may also have been selected in human evolution because of an ability to influence the actions of other MHC molecules and moderate T-cell responses to pathogens. Interestingly, the HLA-A2 and HLA-A3 alleles are derived from two distinct MHC class I lineages dating back to the common ancestor of humans, gorillas and chimpanzees; whereas only one type has survived in each of the ape lineages, humans have retained both. Thus, the HLA-A2 and HLA-A3 alleles must have co-evolved over millions of years, allowing many opportunities for refinement of the beneficial TCR cross-reactivities which might modulate thymic selection to provide enhanced protection against autoimmune diseases such as MS (Lawlor *et al.*, 1991 ▶).

## 4. Concluding remarks

Here, we present the first crystal structure of HLA-A3 in complex with an autoantigenic peptide from a proteolipid protein which is implicated in MS pathogenesis. Analysis of the structure of A3–PLP45-53 and comparison with HLA-A2 reveal a potential role for TCR cross-reactivity with HLA-A3 and A2 to modify T-cell selection and TCR expression *in vivo* (Friese *et al.*, 2008 ▶). Comparison of HLA-A3–PLP45-53 to other HLA-A3-like molecules suggests that subtle alterations in the binding groove of HLA-A3-like molecules may sufficiently alter the presentation of a common peptide to explain different positive and neutral disease associations of HLA-A3-family members.

## Supplementary Material

PDB reference: HLA-A*0301 complex, 2xpg

## Figures and Tables

**Table 1:** Crystallization, data collection, phasing, refinement statistics and model quality Values in parentheses are for the highest resolution shell.

Crystallization	
Protein	6.9 mg ml^−1^ in 10 m*M* HEPES pH 7.0, 10 m*M* NaCl
Reservoir	20% PEG 3350, 0.1 *M* Bis-Tris propane pH 8.0, 0.2 *M* sodium/potassium phosphate (Molecular Dimensions PACT Premier condition No. 94)
Cryoprotectant	25% ethylene glycol
Data collection	
Beamline	I04, Diamond Light Source, Oxfordshire, England
Detector	ADSC Q315
Software	*DENZO*/*SCALEPACK*
Space group	*P*2_1_2_1_2_1_
Unit-cell parameters (Å, °)	*a* = 62.9, *b* = 65.5, *c* = 107.3, α = β = γ = 90
Wavelength (Å)	0.9699
Resolution (Å)	30.0–2.69 (2.81–2.69)
Completeness (%)	100 (100)
Unique reflections	12447 (1223)
Average muliplicity per shell	5.5 (5.7)
〈*I*/σ(*I*)〉	7.87 (1.99)
*R*_merge_†	0.20 (0.94)
Phasing	
Method	Molecular replacement
Software	*MOLREP*
Model used	PDB entry 1duz
Refinement statistics	
Software	*REFMAC*5/*PHENIX*
Resolution (Å)	20–2.7
Completeness (%)	99.41
No. of unique reflections	11859
*R* factor‡ (%)	19.3
*R*_free_§ (%)	25.0
R.m.s.d bond length (Å)	0.006
R.m.s.d. bond angles (°)	0.981
R.m.s.d. chiral volume (Å^3^)	0.067
Average *B* value for main chains (Å^2^)	36.0
Average *B* value for side chains and waters (Å^2^)	40.4
Model quality	
Software	*MolProbity*
Total No. of residues	415
Residues in Ramachandran favoured region (%)	96.0
Ramachandran outliers (%)	0.0
PDB code	2xpg

† *R*_merge_ = 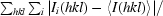 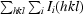, where *hkl* is the unique reflection index, *I_i_*(*hkl*) is the intensity of the symmetry-related reflection and 〈*I*(*hkl*)〉 is the mean intensity.

‡ *R* = 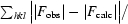 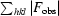, where *hkl* defines the unique reflections.

§ *R*_free_ was calculated over 5.0% of total reflections excluded from refinement.

**Table 2:** List of atomic interactions between HLA-A3 and proteolipid protein (PLP) residues 45–53 (KLIETYFSK)

Peptide		Hydrogen-bond partner†			
Residue	Atom	Residue	Atom	Distance (Å)	Nonbonded contacts‡
Lys (P1)	N	Tyr171	OH	2.70	Glu63, Trp167
	N	Tyr7	OH	2.84	
	O	Tyr59	OH	2.59	
Leu (P2)	N	Glu63	OE1	2.77	Tyr7, Met45, Asn66, Val67
Ile (P3)	N	Tyr99	OH	3.13	Tyr99, Tyr159
Tyr (P6)	OH	Asn66	O	2.94	Ala69, Gln70, Thr73
Phe (P7)					Trp147, Glu152
Ser (P8)	OG	Lys146	NZ	3.07	
	O	Trp147	NE1	2.92	
Lys (P9)	O	Tyr84	OH	2.78	Asp77, Tyr123, Tyr147
	O	Thr143	OG1	3.14	
	NZ	Asp116 (S§)	OD2	2.50	
	N	Asp77	OD1	2.87	

† The cutoff distance of a hydrogen bond is taken to be 3.2 Å.

‡ Residues involved in nonbonded contacts were defined as contact residues within 4 Å of any PLP45-53 peptide atom.

§ S denotes the existence of a salt bridge.

**Table 3:** Peptide-binding groove polymorphisms between HLA-A2 and the A3 superfamily Adapted from Kubo *et al.* (1994 ▶).

	Residues								Residues						Residues			
	B pocket								F pocket						Central groove			
Allele	9	45	63	66	67	70	99	Anchor residue	74	77	80	81	116	Anchor residue	62	73	97	114
HLA-A2	F	M	E	K	V	H	Y	L, M (Falk *et al.*, 1991 ▶)	D	D	T	L	Y	V (Falk *et al.*, 1991 ▶)	G	T	R	H
HLA-A3	F	M	E	N	V	Q	Y	V, L, M (Kubo *et al.*, 1994 ▶)	D	D	T	L	D	K (Kubo *et al.*, 1994 ▶)	Q	T	I	R
HLA-A11	Y	M	E	N	V	Q	Y	T, V (Kubo *et al.*, 1994 ▶)	D	D	T	L	D	K (Kubo *et al.*, 1994 ▶)	Q	T	I	R
HLA-Aw*68	Y	M	N	N	V	Q	Y	T, V (Guo *et al.*, 1992 ▶)	D	D	T	L	D	R (Guo *et al.*, 1992 ▶)	R	T	M	R
HLA-A33	T	M	N	N	V	H	Y	A, I, L, F, Y, V (Falk *et al.*, 1994 ▶)	D	D	T	L	D	R (Falk *et al.*, 1994 ▶)	R	I	M	Q
HLA-A31	T	M	E	N	V	H	Y	L, V, Y, F (Falk *et al.*, 1994 ▶)	D	D	T	L	D	R (Falk *et al.*, 1994 ▶)	Q	I	M	Q
